# Kainic Acid-Induced Neurotoxicity: Targeting Glial Responses and Glia-Derived Cytokines

**DOI:** 10.2174/157015911795596540

**Published:** 2011-06

**Authors:** Xing-Mei Zhang, Jie Zhu

**Affiliations:** 1 Department of Neurobiology, Care Sciences and Society, Karolinska Institute, Stockholm, Sweden; 2 Department of Neurology, The First Hospital of Jilin University, Changchun, China

**Keywords:** Kainic acid, excitotoxicity, microglia, astrocytes, cytokines.

## Abstract

Glutamate excitotoxicity contributes to a variety of disorders in the central nervous system, which is triggered primarily by excessive Ca^2+^ influx arising from overstimulation of glutamate receptors, followed by disintegration of the endoplasmic reticulum (ER) membrane and ER stress, the generation and detoxification of reactive oxygen species as well as mitochondrial dysfunction, leading to neuronal apoptosis and necrosis. Kainic acid (KA), a potent agonist to the α-amino-3-hydroxy-5-methyl-4-isoxazolepropionic acid (AMPA)/kainate class of glutamate receptors, is 30-fold more potent in neuro-toxicity than glutamate. In rodents, KA injection resulted in recurrent seizures, behavioral changes and subsequent degeneration of selective populations of neurons in the brain, which has been widely used as a model to study the mechanisms of neurodegenerative pathways induced by excitatory neurotransmitter. Microglial activation and astrocytes proliferation are the other characteristics of KA-induced neurodegeneration. The cytokines and other inflammatory molecules secreted by activated glia cells can modify the outcome of disease progression. Thus, anti-oxidant and anti-inflammatory treatment could attenuate or prevent KA-induced neurodegeneration. In this review, we summarized updated experimental data with regard to the KA-induced neurotoxicity in the brain and emphasized glial responses and glia-oriented cytokines, tumor necrosis factor-α, interleukin (IL)-1, IL-12 and IL-18.

## INTRODUCTION

Excitotoxicity mediated by glutamate receptors may underlay the pathology of a number of neurological abnormalities, including Huntington’s chorea, Alzheimer’s disease (AD), and Parkinson’s disease (PD). Excitotoxic cell death is commonly induced experimentally by the administration of kainic acid (KA), a potent agonist to the α-amino-3-hydroxy-5-methyl-4-isoxazolepropionic acid (AMPA)/kainate class of glutamate receptors [1-3]. In rodents, injections of KA resulted in recurrent seizures, behavioral changes and subsequent degeneration of selective populations of neurons in the brain [4, 5]. Thus, administration of KA in rodents has been widely used as a model to study the mechanisms of neurodegenerative pathways induced by excitatory neurotransmitters.

## 1. OVERVIEW OF EXCITOTOXICITY INDUCED BY GLUTAMATE

L-glutamate, the major excitatory transmitter in the brain and spinal cord, is associated with learning, cognition, memory and neuro-endocrine functions [6, 7]. The glutamate receptors can be divided into two broad categories: the ionotropic receptors that mediate fast postsynaptic potentials by activating ion channels directly, and the metabotropic receptors that results in the expression of slow postsynaptic potentials through second messengers [8, 9]. The action of glutamate on the ionotropic receptors is always excitatory [10, 11]. There are three major subtypes of ionotropic glutamate receptors: AMPA, kainate, and N-methyl-D-aspartate (NMDA), named according to the types of synthetic agonists that activate them, respectively [12, 13]. The NMDA glutamate receptor is blocked by specific antagonists such as D(-)-2-amino-5-phosphonovalerate (APV) [14, 15]. Both AMPA and kainate receptors are blocked by 6-cyano-7-nitroquinoxalin-2,3-dione (CNQX) [16, 17]. Thus the AMPA and kainate receptors are sometimes referred to together as non-NMDA receptors. The ion channel of NMDA receptor is a tetrameric structure that results from up to seven genes coding for seven subunits termed GluN1, GluN2A, GluN2B, GluN2C, GluN2D, GluN3A and GluN3B [18, 19]. The AMPA receptor family is composed of four subunits, GluA1–4 [20, 21]. The kainate receptor family comprises five genes, divided into two subfamilies, including GluK4-5 and GluK1–3. GluK4 and GluK5 exhibit higher affinity for kainate than GluK1–3 [22, 23]. Herein, we used the new nomenclature for glutamate receptors recommended by the International Union of Pharmacology Committee on Receptor Nomenclature and Drug Classification (NC-IUPHAR) [24]. NC-IUPHAR recommended and previous nomenclatures of ionotropic gluatamate receptor subunits are listed in Table **1**.

Excessive amounts of glutamate are highly toxic to neurons, an action termed glutamate excitotoxicity [25, 26]. Glutamate excitotoxicity is triggered primarily by excessive Ca^2+^ influx arising from overstimulation of the NMDA subtype of glutamate receptors, followed by disintegration of the endoplasmic reticulum (ER) membrane and ER stress, the generation of reactive oxygen species (ROS) as well as mitochondrial dysfunction, leading to neuronal apoptosis and necrosis [27, 28]. There is increasing realization that the mitochondrial dysfunction occupies the center stage in these processes [28, 29]. In many cell types, glutamate neurotoxicity is induced by NMDA as well as non-NMDA receptors [25, 26, 30].

## 2. KA ADMINISTRATION TO RODENTS INDUCES SEIZURES, SELECTIVE NEURODEGENERATION AND BEHAVIORAL CHANGES

Kainic acid (KA) is a non-degradable analog of glutamate and 30-fold more potent in neurotoxicity than glutamate [31-33]. Administration of KA to rodents caused a well characterized seizure syndrome, as described by Ben-Ari and other research groups [34, 35]. The seizure activity caused by intravenous, intraperitoneal, intranasal injections or microinjection into amygdala or hippocampus is divided in several distinct phases. During the first 20-30 min, the animals have “staring” spells, followed by head nodding and numerous wet-dog shakes for another 30 min. One hour after KA administration, the animal starts recurrent limbic motor seizures, including masticatory and facial movements, forepaws tremor, rearing and loss of postural control. The seizures then become progressively severer, with a reduction in the intermission. In the following l-2 h, the animal displays a full status epilepticus [34-36].

**Fig. (1):**
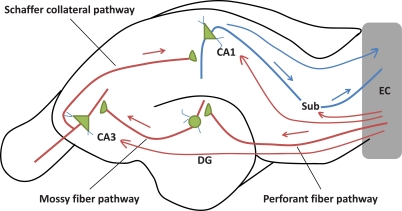
**The Input and Output Pathways of Hippocampal Formation.** Entorhinal cortex (EC) is the main input to the hippocampus. EC projects to the dentate gyrus (DG) *via* perforant fiber pathway and provides the critical input to CA3 *via* mossy fiber pathway, then to CA1 by means of the Schaffer collateral pathway. Additionally, EC can also project directly to CA3, CA1 and subiculum (Sub). Meantime, EC is the major output of the hippocampus. Arrows denote the direction of impulse flow.

KA-induced damage seriously impact the hippocampus. The hippocampus is particularly vulnerable to KA-induced neurotoxicity due to the high density of kainate receptors [37]. The hilar neurons are sensitive to KA-induced neurotoxicity, but neuron loss in the other areas of the hippocampus differs between animal species and strains [38-40]. In rats, the systemic injections of KA produced widespread neuronal death, primarily in the hippocampus hilus, CA1 and CA3 areas [30, 41]. Mouse strains vary significantly in their sensitivity to KA-induced neurodegeneration [39, 40, 42, 43]. In general, the C57BL/6, C57BL/10, and (C57BL/6 x CBA/J) F1 strains are resistant to KA-induced neurodegeneration, while the FVB/N, ICR and DBA/2 J strains are vulnerable [40]. C57BL/6, the “relatively” resistant mouse strain, reveals significant neuronal damage in CA1 and CA3, and to a lesser extent, in the polymorphic layer of the dentate gyrus 12 h post-treatment of KA systemically detected by cupric-silver and Fluoro-Jade B staining [44, 45]. CA3 region has the highest abundance of kainate receptors, the activation of which can elevate the concentration of ROS and impair the normal function of mitochondria [46-48]. CA3 neurons are directly excited by stimulation of their KA receptors and indirectly, by increased glutamate efflux secondary to KA stimulation of mossy fibers [49, 50]. CA3 synchronization produces spreading epileptiform activity that extends to CA1 and other limbic structures [51, 52] (Fig. **1**). 

CA1 pyramidal neurons receive two distinct excitatory inputs that are capable of influencing hippocampal output and involving in spatial memory and memory consolidation [53, 54]. Damage in CA1/CA3 regions of hippocampus induced by KA mainly results in the spatial learning deficits [55, 56]. KA-treated Wistar rats are impaired in the water maze and object exploration tasks, and hyperactive in the open field test, which can be improved by physical exercise and the selective cyclooxygenase (COX)-2 inhibitor [57, 58]. Intraperitoneal injections of KA into the developing rat brains induce the impaired short-term spatial memory in the radial-arm maze, deficient long-term spatial learning and retrieval in the water maze, and a greater degree of anxiety in the elevated plus maze [59, 60]. Mice with a single unilateral injection of KA into the dorsal hippocampus exhibit a decrease in depression-like behavior in the forced swimming test and retarded acquisition as well as impaired retention of visual-spatial information in the Morris water maze test [61].

## 3. KA MEDIATES GENERATION OF OXIDATIVE STRESS

**Fig. (2):**
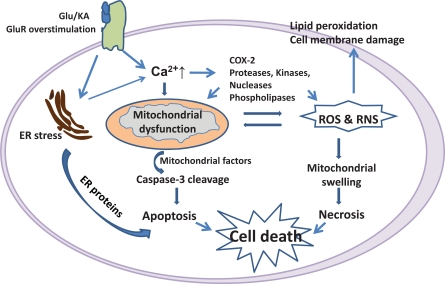
**Schematic Overview of KA-Mediated Neuronal Death.** (1) By stimulating glutamate receptors (GluR), kainic acid (KA) elicits the increase of intracellular Ca^2+^, activation of Ca^2+^-dependent enzyme and production of free radicals; (2) Excessive Ca^2+^ and free radicals cause mitochondrial dysfunction, release of mitochondrial factors, activation of caspase-3, leading to neuronal apoptosis; (3) KA causes the disintegration of the endoplasmic reticulum (ER) and ER stress with the activation of the ER proteins Bip, Chop, and caspase-12, involved in neuronal apoptosis; (4) Ca^2+^ overload and excessive free radicals cause directly mitochondrial swelling, leading to neuronal necrosis. COX: cyclooxygenase; ROS: reactive oxygen species; RNS: reactive nitrogen species.

KA receptors have both presynaptic modulatory and direct postsynaptic excitatory actions [62, 63]. The activation of KA receptors produces membrane depolarization and results in alteration in intracellular calcium concentrations, which is required to trigger the neuronal death cascade (Fig. **2**) [64]. KA can also induce the release of lactate dehydrogenase (LDH), and a decrease in 3-(4, 5-dimethylthiazole-2-yl)-2, 5-diphenyl tetrazolium bromide (MTT), which result in damage of mitochondrial function [2]. KA administration increases the generation of ROS and reactive nitrogen species (RNS). There is growing evidence that free radical generation plays a key role in the neuronal damage [65]. KA has been shown to immediately induce COX-2 expression that might be involved in hippocampal neuronal death [66]. Early induced COX-2 facilitates the recurrence of hippocampal seizures, and late synthesized COX-2 stimulates hippocampal neuron loss after KA administration [67]. COX catalyzes the first step in the synthesis of prostanoids, including prostaglandins (PGs), prostacyclin, and thromboxanes. PGE(2) is pathologically increased in the brain after KA treatment, and has been proven to be closely associated with neuronal death [68]. In addition, lipid peroxides play critical roles in the initiation and modulation of inflammation and oxidative stress upon KA insult. Seizures can induce the products of lipid peroxidation, such as F(2)-isoprostanes and Isofurans, which have been thought to be the reliable indices of oxidative stress *in vivo* [69]. Moreover, KA causes the disintegration of the ER membrane in hippocampal neurons and ER stress with the activation of the ER proteins Bip, Chop, and caspase-12 [70]. ER stress appears to act at an early stage of the cell death process prior to disruption of calcium homeostasis, excessive accumulation of ROS, and mitochondrial dysfunction [71]. Old astrocyte specifically induced substance (OASIS) is involved in the endoplasmic reticulum stress response [72]. A recent study showed that OASIS expressed in astrocytes plays an important role in protection against neuronal damage induced by KA [1].

## 4. GLIAL CELLS ARE ACTIVATED UPON KA ADMINISTRATION

**Fig. (3):**
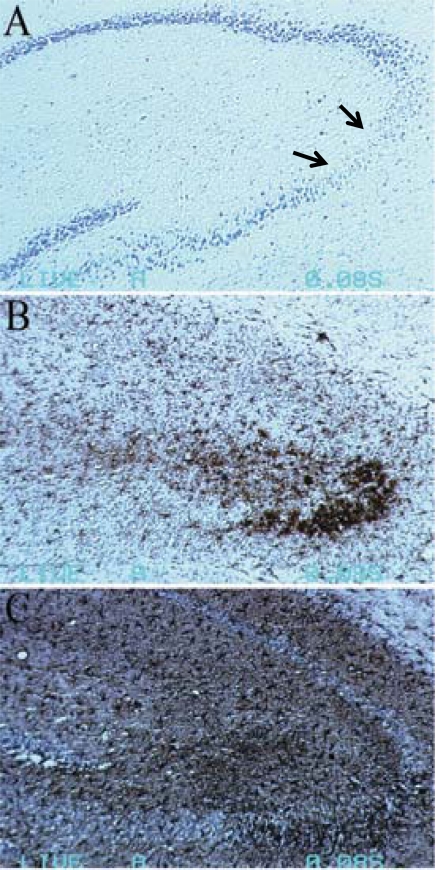
**Glial cells activation accompanied the neuronal death 7 days after KA (45 mg/kg body weight) treatment to C57BL/6 mice.** (**A**) Obvious neuronal loss was showed in CA3 area of hippocampus by Nissl’s staining. (**B**) CD11b positive cells (microglia) accumulated in the lesioned CA3 area. (**C**) GFAP positive cells (astrocytes) spread the whole hippocampus, especially in CA3 area. Arrows in A indicate the areas of neuronal loss.

KA-induced neuronal death is accompanied by increased activation of microglia and astrocytes [73-75]. Additionally, the activated glial cells cluster at the hippocampal lesions and the immunostaining reactivity is particularly strong around areas of debris (Fig. **3**). 

### 4.1. Microglia

**Fig. (4):**
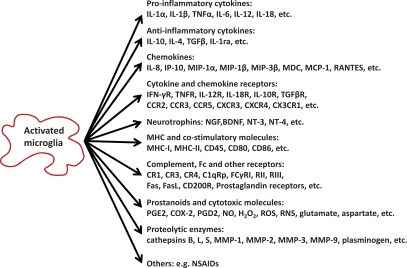
**The Categories of Molecules Produced by Activated Microglia.** IL: interleukin; TNF: tumor necrosis factor; TGF: transforming growth factor; IL-1ra: IL-1 receptor antagonist; MIP: macrophage inflammatory protein; MDC: macrophage-derived chemokine; MCP: monocyte-chemoattractant protein; RANTES: regulated on activation normal T cell expressed and secreted; IFN: interferon; IP: IFN-inducible protein; R: receptor; NGF: nerve growth factor; BDNF: brain-derived neurotrophic factor; NT: neurotrophin; MHC: major histocompatibility complex; CR: complement receptor; C1qRp: C1q receptor for phagocytosis enhancement; FasL: Fas ligand; PG: prostaglandin; COX: cyclooxygenase; NO: nitric oxide; ROS: reactive oxygen species; RNS: reactive nitrogen species; MMP: matrix metalloproteinase; NSAIDs: nonsteroidal antiinflammatory drugs.

Microglia account for approximately 20% of the total glial population in the central nervous system (CNS). Microglia are the main effector cell type of the immune and inflammatory responses in the CNS, as earlier reviewed by Streit and his colleagues [76]. The normal role of microglia could be partly connected to neuroprotection, whereas in pathological conditions microglia may become disease-promoting cells. Upon neuronal injury, microglia rapidly acquire changes in morphology and secrete a variety of soluble mediators [77, 78] (Fig. **4**). Some studies suggested that the activated microglia might exert a neuroprotective function, especially in multiple sclerosis (MS) and its animal model, experimental autoimmune encephalomyelitis (EAE) by creating a microenvironment for reparative and regenerative processes [79]. Evidence is also accumulating that activated microglia induce and/or exacerbate neuropathological changes in several CNS diseases such as AD and PD through secreting proinflammatory and neurotoxic factors [80, 81]. In KA-induced hippocampal injury, microglial activation is generally believed to contribute to neuroinflammation and neurodegeneration [74, 82, 83]. A recent study showed that IκB kinase/nuclear factor kappa B (NF-κB) dependent microglial activation participated in KA-mediated injury *in vivo* through induction of inflammatory mediators [82]. However, whether microglial activation initiates the disease progression or merely responds to neuronal death is still unclear. 

### 4.2. Astrocytes

**Fig. (5):**
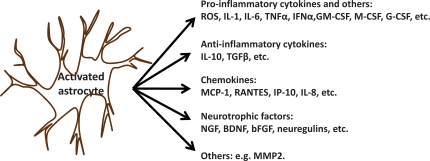
**The Categories of Molecules Produced by Activated Astrocytes.** ROS: reactive oxygen species; IL: interleukin; TNF: tumor necrosis factor; IFN: interferon; CSF: colony-stimulating factor; GM-CSF: granulocyte-macrophage CSF; TGF: transforming growth factor; MCP: monocyte-chemoattractant protein; RANTES: regulated on activation normal T cell expressed and secreted; IP: IFN-inducible protein; NGF: nerve growth factor; BDNF: brain-derived neurotrophic factor; bFGF: basic fibroblast growth factor; MMP: matrix metalloproteinase.

Astrocytes, the most numerous glia cells, have been regarded as passive supporters of neurons in CNS for decades. Studies of the last 20 years, however, challenged this assumption by demonstrating that astrocytes possess functional neurotransmitter receptors [84, 85]. These findings have led to a new concept of neuron-glia intercommunication where astrocytes play an undoubted dynamic role by integrating neuronal inputs and modulating synaptic activity, and so contribute to disease development [86]. Astrocytes have functional receptors for the excitatory neurotransmitter glutamate and respond to physiological concentrations of this substance with oscillations in intracellular Ca^2+^ concentrations and spatially propagating Ca^2+^ signals [87-89]. *In vitro* studies provided evidence that astrocytes can take up glutamate at synapses and release glutamate in a calcium-dependent manner [90]. A proliferative response of astrocytes at two days after KA treatment has been reported already in 1981 [91]. The expression of glial fibrillary acidic protein (GFAP) has been shown to steadily increase from one/three days up to one month after intra-hippocampal or intraperitoneal injection of KA [92, 93]. Astrogliosis induced by excitotoxicity has been considered as a marker for neurotoxicity [73, 94]. Activated astrocytes produce pro- and anti-inflammatory cytokines, chemokines, neurotrophic factors and other modulators to be involved in neuron-glia communication (Fig. **5**). It is believed that astrocytes produce growth factors to prevent neurons from death and to promote proliferation and differentiation of precursor cells [95-97]. Activation of transcription factors, including nuclear factor erythroid-2-related factor 2 (Nrf2) and NF-κB, in astrocytes induces the neuroprotective molecule expression and confers protection to neighboring neurons [98-100]. 

## 5. ALTERED CYTOKINE EXPRESSION AFFECTS KA-INDUCED INJURY

**Fig. (6):**
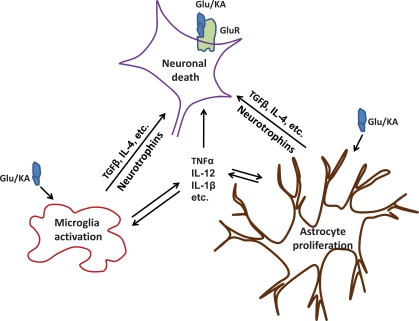
**Cytokines Involved in Neuron-glia Intercommunication.** KA administration enhances further release of endogenous excitatory amino acids, activates microglia and astrocytes. Activated glial cells secrete inflammatory molecules, e.g. cytokines, chemokines, and neurotrophins to influence the outcome of neuronal damage. Glu/KA: glutamate/kainic acid; GluR: glutamate receptors; TGF: transforming growth factor; IL: interleukin; TNF: tumor necrosis factor.

Altered expression of cytokines in response to brain injury has diverse actions that can exacerbate, mediate, reduce or inhibit neuronal damage and influence the disease development in a variety of CNS disorders, such as AD, MS, viral or bacterial infections, ischemia, stroke, and various forms of encephalopathies [101-105]. Cytokines can be divided into pro-inflammatory and anti-inflammatory cytokines, which play the neurodestructive and neuroprotective roles, respectively. It is the balance between destructive and protective factors that ultimately determines the net result of the neuro-immune and neuro-inflammation interaction, as reviewed by Kerschensteiner *et al*. [106]. Results from studies using KA model also indicated that cytokines are involved in neuron-glia intercommunication and manipulation of pro- and anti-inflammatory cytokines can modify the outcome with regard to the seizure activity, behavioral changes as well as the neuropathological consequences [75, 107-109] (Fig. **6**). The roles of several important cytokines in the CNS disorders are reviewed here, including tumor necrosis factor-α (TNF-α), interleukin-1 (IL-1), IL-12 and IL-18.

### 5.1. TNF-α

Tumor necrosis factor-α (TNF-α) is mainly produced by microglia and astrocytes in the CNS. Its functions are mediated through two receptors, TNF receptor (TNFR) 1 (p55) and TNFR2 (p75), both of which are expressed on various cells types [110]. TNF-α over-expression participates in the pathogenesis of several CNS disorders, such as AD [111], bacterial meningitis [112], MS [113] and cerebral malaria [114]. TNF-α potentiates excitotoxic injury to human fetal brain cells [115]. In contrast to its well known deleterious roles, multiple lines of evidence suggested that TNF-α also exhibit neuroprotective properties. This implies an intricate biological function of TNF-α in modulating immune and inflammatory responses. TNF-α knockout worsens Listeria infection in the CNS [116] and TNF-α receptor knockout enhances the neuronal damage after excitotoxic [108, 117], ischemic [118] or traumatic injury [119]. Our study showed that mice lacking TNFR1 exhibited a more severe seizure activity, hippocampal neurodegeneration and increased microglial activation, suggesting that TNF*-*α plays its protective role through TNFR1 signaling [108], which is in agreement with a previous report [120]. Another study also proved that the protective roles of TNF*-*α in KA-induced neurodegeneration are *via* TNFR2 signaling [117]. Several neuroprotective molecules were identified as TNFR1 targets, including members of the Bcl-2 family, DNA repair machinery and cell cycle, developmental, and differentiation factors, neurotransmitters and growth factors, as well as their receptors [121]. The mechanisms by which TNF reduced neuron loss after brain injury may involve the up-regulation of proteins, such as neuronal apoptosis inhibitor protein (NAIP), which maintain calcium homeostasis and reduce free radical generation [122]. 

### 5.2. IL-1

IL-1 plays a pivotal role in the neuroinflammation that has been well addressed in KA induced neurodegenerative model [109, 123]. Systemic KA administration induced the expression of IL-1β, IL-1 receptor antagonist and IL-1β converting enzyme as early as 3 h, 12 h, and 24 h post-treatment, respectively, which is localized in the areas known to display neuronal and tissue damage upon excitotoxic lesions [124, 125]. Recombinant IL-1 receptor antagonist has dose- and region-dependent effects on neuronal survival after KA treatment and prevents damage-induced changes in amyloid precursor protein and GFAP mRNAs [126]. It has also been shown that IL-1β is activated in the cerebellum with systemic administration of KA, and its type I receptor (IL-1R) is expressed at a soma of cerebellar Purkinje cells [127]. The proconvulsive actions of IL-1β in the hippocampus may depend on the activation of a sphingomyelinase- and Src-family of kinases-dependent pathway which leads to the phosphorylation of the GluN2B subunit [128]. 

### 5.3. IL-12

IL-12 is a heterodimeric cytokine that consists of a heavy chain, p40, and a light chain, p35 [129]. In the CNS, microglia and astrocytes are the main source of producing IL-12 [130]. Human CNS-derived microglia produce IL-12 *in vitro* after activation with LPS and IFN-γ [131]. Murine microglia can be induced to express mRNA encoding the IL-12 receptor (IL-12R) [132], indicating an autocrine regulation pathway of IL-12 in these cells. IL-12 plays a critical role in several CNS diseases, such as MS and Borna disease. IL-12p40 is increased in cerebrospinal fluid and serum of MS patients [133]. Mice lacking IL-12p40 or administered with anti-IL-12p40 monoclonal antibodies are resistant to the development of EAE [134, 135]. In contrast, IL-12p35 deficient mice are susceptible to EAE [114]. Borna disease virus, which does not trigger disease in most strains, is harmful after infecting the CNS of mice that overexpress IL-12, suggesting an important role of IL-12 in viral infection of the CNS [136]. IL-12 is an active participant in excitotoxic brain injury as suggested by our previous observation that IL-12p35 deficiency alleviates KA-induced hippocampal neurodegeneration [107] and by another finding that IL-12 expression is reduced in hippocampi of transgenic mice protected against KA-induced excitotoxicity by metallothionein overexpression [137]. 

### 5.4. IL-18

Interleukin (IL)-18 is most closely related to IL-1β. The similarities between both cytokines comprise structure, receptor complex, and pro-inflammatory properties [138]. IL-18 serves as a link between innate and adaptive immune responses, such as stimulating the expression of adhesion molecules, inducing the production of chemokines (IL-8) and cytokines (TNF-α and IL-6), stimulating the activity of NK cells, and promoting T helper 1 (Th1) cells responses in combination with IL-12, Th2 responses in combination with IL-4, and Th17 responses in combination with IL-23 [139]. IL-18 and IL-18 receptor (IL-18R) mRNA have been found in brain tissue and in cultured astrocytes and microglia [140]. IL-18 enhanced postsynaptic AMPA receptor responses in CA1 pyramidal neurons *via* the release of glutamate, thereby facilitating basal hippocampal synaptic transmission [141]. IL-18 deficient mice showed a diminished microglial activation and reduced dopaminergic neuron loss after acute 1-methy-4-phenyl-1,2,3,6-tetrahydropyridine treatment [142]. The roles of IL-18 in KA-induced model are controversial. Levels of IL-18 and IL-18R in hippocampus increase progressively from day 1 and peaked at day 3 post-KA treatment [143]. Interestingly, intracerebellar coinjection of IL-18 counteracts the effect of IL-1β in KA-induced ataxia in mice [144]. We showed that exogenous IL-18 administration aggravated the KA-induced injury in normal C57BL/6 mice, while in the condition of IL-18 deficiency, IL-12 could overcompensate the function of IL-18 and worsen the seizure activity as well as hippocampal neurodegeneration [75]. 

## 6. THERAPEUTIC STRATEGIES

**Fig. (7):**
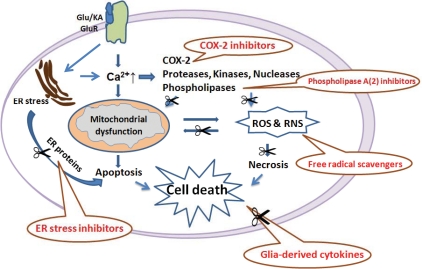
**Schematic Illustration of Anti-Oxidant and Anti-inflammatory Treatments.** By inhibiting one of the major components of the neuroinflammatory response after KA treatment, there could be less inflammation and neuronal loss. The potential treatments include (1) cyclooxygenase (COX)-2 inhibitors and other antioxidants; (2) Phospholipase A2 inhibitors; (3) Free radical scavengers; (4) endoplasmic reticulum (ER) stress inhibitors; and (5) Glia-derived cytokines. Glu/KA: glutamate/kainic acid; GluR: glutamate receptors; ROS: reactive oxygen species; RNS: reactive nitrogen species.

Considering that oxidative stress is central to KA-induced excitotoxic damage, anti-oxidant and anti-inflammatory treatments may attenuate or prevent the KA-mediated neurodegeneration (Fig. **7**). The potential role of COX-2 inhibitors as a new therapeutic drug for the neuron loss after KA treatment has been studied. The selective COX-2 inhibitors, celecoxib, NS398, rofecoxib and SC58125, can suppress an elevation of PGE (2) and block hippocampal cell death [57, 145]. The other drugs tested experimentally include curcumin, fluoxetine, ethyl pyruvate and statins, whose neuroprotective effects are associated with their anti-inflammatory and anti-oxidant effects [146]. Free radical scavengers are well known to prevent neuron loss induced by exposure to excitotoxins. Edaravone (Ed), a newly developed free radical scavenger, could inhibit lipid peroxidation and prevent neuron loss when administered after the onset of seizures in a KA-induced neurodegenerative animal model [147]. The pineal secretory product, melatonin, has free-radical-scavenger and antioxidant properties, which attenuates KA-induced neuronal death, lipid peroxidation, and microglial activation [148]. Several phospholipase A (2) inhibitors, quinacrine and chloroquine, arachidonyl trifluoromethyl ketone, bromoenol lactone, cytidine 5-diphosphoamines, and vitamin E, have been shown to prevent the neurodegeneration in KA-mediated neurotoxicity [149]. Moreover, inhibition of ER stress by small molecular compounds, such as salubrinal, may benefit for treatment of KA-mediated neurotoxicity [70]. Furthermore, the microtubule interacting drug candidate NAP has been shown to protect against KA toxicity *via* regulating the expression of key genes involved in the epileptogenic pathway [150]. A novel approach to engineer patient derived adult stem cells for therapeutic adenosine delivery in autologous cell transplantation has been proved to suppress seizure activity and protect hippocampal neurons from KA-induced damage [151]. Additionally, targeting the pro-inflammatory cytokines, by blocking the unique signal transduction of the specific cytokine is another potential therapeutic strategy. 

## 7. CONCLUDING REMARKS

Glutamate excitotoxicity contributes to a variety of CNS diseases, which is involved in overstimulation of glutamate receptors, excessive Ca^2+^ influx, ER stress, generation of ROS and mitochondrial dysfunction, leading to neuronal damage. KA is a potent agonist to the AMPA/kainate class of glutamate receptors, which can result in seizures, behavioral changes and neurodegenration in susceptible brain regions like the hippocampus in rodents.

The activated glia cells and subsequent secretion of inflammatory molecules can modify the outcome of disease progression. By inhibiting one of the major components of the neuroinflammatory response after KA treatment, there could be less inflammation and neuronal loss, therefore an improvement of cognitive function. 

## Figures and Tables

**Table 1:** NC-IUPHAR Recommended and Previous Nomenclatures of Ionotropic Glutamate Receptor Subunits

Ionotropic Glutamate Family	NC-IUPHAR Subunit Nomenclature	Previous Nomenclatures	Human Gene Name	Human Chromosomal Location
NMDA	GluN1	GLU_N1_, NMDA-R1, NR1, GluRξ1	GRIN1	9q34.3
	GluN2A	GLU_N2A_, NMDA-R2A, NR2A, GluRε1	GRIN2A	16p13.2
	GluN2B	GLU_N2B_, NMDA-R2B, NR2B, hNR3, GluRε2	GRIN2B	12p12
	GluN2C	GLU_N2C_, NMDA-R2C, NR2C, GluRε3	GRIN2C	17q25
	GluN2D	GLU_N2D_, NMDA-R2D, NR2D, GluRε4	GRIN2D	19q13.1
	GluN3A	GLU_N3A_, NMDA-R3A, NMDAR-L, chi-1	GRIN3A	9q31.1
	GluN3B	GLU_N3B_, NMDA-R3B,	GRIN3B	19p13.3
AMPA	GluA1	GLU_A1_, GluR1, GluRA, GluR-A, GluR-K1, HBGR1	GRIA1	5q31.1
	GluA2	GLU_A2_, GluR2, GluRB, GluR-B, GluR-K2, HBGR2	GRIA2	4q32-q33
	GluA3	GLU_A3_, GluR3, GluRC, GluR-C, GluR-K3	GRIA3	Xq25-q26
	GluA4	GLU_A4_, GluR4, GluRD, GluR-D	GRIA4	11q22
Kainate	GluK1	GLU_K5_, GluR5, GluR-5, EAA3	GRIK1	21q22.11
	GluK2	GLU_K6_, GluR6, GluR-6, EAA4	GRIK2	6q16.3-q21
	GluK3	GLU_K7_, GluR7, GluR-7, EAA5	GRIK3	1p34-p33
	GluK4	GLU_K1_, KA1, KA-1, EAA1	GRIK4	11q22.3
	GluK5	GLU_K2_, KA2, KA-2, EAA2	GRIK5	19q13.2
